# 2151. *In Vitro* Activity of Ceftazidime-Avibactam and Comparator Agents Against *Pseudomonas aeruginosa* Collected from Patients with Presumed Hospital- and Community-Acquired Respiratory Tract Infections: ATLAS Global Surveillance Program, 2017-2021

**DOI:** 10.1093/ofid/ofad500.1774

**Published:** 2023-11-27

**Authors:** Mark G Wise, Gregory Stone, Daniel F Sahm

**Affiliations:** IHMA, Schaumburg, Illinois; Pfizer, Inc., Groton, Connecticut; IHMA, Schaumburg, Illinois

## Abstract

**Background:**

The β-lactamase inhibitor, avibactam, has potent inhibitory activity against Class A, Class C, and certain Class D serine β-lactamases. This study evaluated the *in vitro* activity of ceftazidime-avibactam (CZA) and comparators against respiratory tract infection (RTI)-associated *Pseudomonas aeruginosa* isolated from presumed community-acquired (CA; cultured < 48 hours after hospital admission) and hospital-acquired (HA; cultured ≥48 hours post-admission) infections collected from 2017-2021.

**Methods:**

11,780 non-duplicate *P. aeruginosa* were collected from 246 sites in 54 countries as part of ATLAS 2017-2021 (excluding mainland China and North America) from respiratory tract infections for which the length of hospitalization was specified. Susceptibility testing was by broth microdilution according to CLSI guidelines and analyzed using CLSI 2023 breakpoints. Meropenem-nonsusceptible (MEM-NS) isolates were screened for the presence of β-lactamase genes. All MEM-NS isolates were screened in 2017-2020, whereas ∼25% of those collected in 2020 and 2021 were screened.

**Results:**

CZA was the most active agent examined against the CA-infection isolates, inhibiting 92.3% of the population (Table). Against the HA isolates, 89.0% were susceptible to CZA, a percentage slightly lower than amikacin (89.4%). Removing MBL-producers increased the percentages susceptible to CZA for both groups by 2-3 percentage points. Approximately 68-70% of the meropenem-nonsusceptible isolates from both groups were susceptible to CZA, values similar to those demonstrated by amikacin and ceftolozane/tazobactam.

**Figure:**
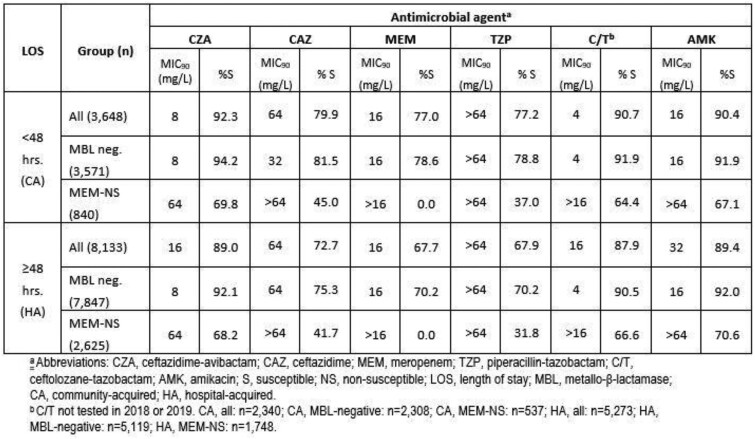

**Conclusion:**

The percentage of CA and HA isolates from respiratory tract infections inhibited by CZA was approximately 15 and 20 percentage points higher, respectively, than the percentages inhibited by meropenem, and slightly higher (1-2 percentage points) than ceftolozane-tazobactam. The potent *in vitro* activity of CZA against HA isolates was exceeded only by amikacin, an agent discouraged as monotherapy for *P. aeruginosa* infections outside of the urinary tract by the CLSI. CZA remains an excellent therapeutic choice for use against *P. aeruginosa*, regardless of whether they’re CA or HA.

**Disclosures:**

**Mark G Wise, PhD**, Merck & Co., Inc.: Honoraria|Pfizer Inc.: Honoraria|Venatorx: Paid fees for conducting the study and abstract preparation **Gregory Stone, PhD**, Pfizer: Stocks/Bonds **Daniel F. Sahm, PhD**, Merck & Co., Inc.: Honoraria|Pfizer Inc.: Honoraria|Venatorx: Paid fees for conducting the study and abstract preparation

